# Treatment of polyethylene microplastics degraded by ultraviolet light irradiation causes lysosome-deregulated cell death

**DOI:** 10.1038/s41598-024-74800-y

**Published:** 2024-10-14

**Authors:** Sota Manabe, Yuya Haga, Hirofumi Tsujino, Yudai Ikuno, Haruyasu Asahara, Kazuma Higashisaka, Yasuo Tsutsumi

**Affiliations:** 1 School of Pharmaceutical Sciences, Osaka University, 1-6 Yamadaoka, Suita, Osaka 565-0871 Japan; 2 Graduate School of Pharmaceutical Sciences, Osaka University, 1-6 Yamadaoka, Suita, Osaka 565-0871 Japan; 3 Museum Links, Osaka University, 1-13 Machikaneyama, Toyonaka, Osaka 560-0043 Japan; 4 Institute for Open and Transdisciplinary Research Initiatives, Osaka University, 1-1 Yamadaoka, Suita, Osaka 565-0871 Japan; 5 Institute for Advanced Co-Creation Studies, Osaka University, 1-6 Yamadaoka, Suita, Osaka 565-0871 Japan; 6 Global Center for Medical Engineering and Informatics, Osaka University, 2-2 Yamadaoka, Suita, Osaka 565-0871 Japan

**Keywords:** Autophagy, Lysosome dysregulation, Microplastics, Polyethylene microplastics, Surface degradation of microplastics, Cell death, Environmental impact

## Abstract

**Background:**

Microplastics (MPs), plastic particles < 5 mm in size, are prevalent in the environment, and human exposure to them is inevitable. To assess the potential risk of MPs on human health, it is essential to consider the physicochemical properties of environmental MPs, including polymer types, size, shape, and surface chemical modifications. Notably, environmental MPs undergo degradation due to external factors such as ultraviolet (UV) rays and waves, leading to changes in their surface characteristics. However, limited knowledge exists regarding the health effects of MPs, with a specific focus on their surface degradation. This study concentrates on cytotoxic MPs with surface degradation through UV irradiation, aiming to identify the mechanisms underlying their cell toxicity.

**Results:**

Polyethylene (PE) and surface-degraded PE achieved through UV light irradiation were employed as model MPs in this study. We explored the impact of PE and degraded PE on cell death in murine macrophage cell line RAW264.7 cells and human monocyte cell line THP-1 cells. Flow cytometric analysis revealed that degraded PE induced programmed cell death without activating caspase 3, while non-degraded PE did not trigger programmed cell death. These findings suggest that degraded PE might induce programmed cell death through mechanisms other than caspase-driven apoptosis. To understand the mechanisms of cell death, we investigated how cells responded to degraded PE-induced cellular stress. Immunofluorescence and western blotting analyses demonstrated that degraded PE induced autophagosome formation and increased p62 expression, indicating inhibited autophagy flux after exposure to degraded PE. Furthermore, degraded PE exposure led to a decrease in acidic lysosomes, indicating lysosomal dysregulation. These results imply that degraded PE induces lysosomal dysfunction, subsequently causing autophagy dysregulation and cell death.

**Conclusions:**

This study unveils that UV-induced degradation of PE results in cell death attributed to lysosomal dysfunction. The findings presented herein provide novel insights into the effects of surface-degraded MPs on biological responses.

## Background

Microplastics (MPs) are generally defined as plastic particles < 5 mm in size that are widely present in the environment^1^. MPs are highly stable and do not decompose naturally and remain in the environment^2^ As a result, MPs are ubiquitous in various environments, including in ocean, air, and soil^3,4^. Humans cannot avoid exposure to MPs through ingestion, inhalation, or skin contact^5^. In fact, MPs were detected in human blood, human placenta, and human lung^6–8^. Consequently, there is growing concern regarding the potential impact of MPs on human health. To investigate and evaluate the potential risk of MPs, it is crucial to emphasize that MPs in the environment exhibit complex physicochemical properties, including a wide variety of compositions, shapes, and chemical surface modifications^9^. Hence, understanding these complexities is vital for a comprehensive assessment of the potential implications of MPs on human health. However, little is known about whether MPs are safe for human health and how the complex physicochemical properties of MPs in the environment affect their safety.

Although several studies assessed cytotoxic effect of MPs, how MPs have an impact on cytotoxic response is not fully understood. Polystyrene microplastics (PSMPs) induce pulmonary cytotoxicity by generating reactive oxygen species. Moreover, PSMPs cause inflammation in the conjunctiva and lacrimal glands^10^. Polyethylene microplastics (PEMPs) co-exposed with tetrabromobisphenol A (TBBPA) exacerbate cytotoxicity; however, its sole effect is weak. In addition, changes in gut microbiota caused by PEMPs and TBBPA may disrupt gut homeostasis^11^. In order to evaluate the potential impact on biological response, it is crucial to consider the physicochemical properties of MPs. MPs in the environment is heterogenous in terms of polymer types, size, shape, and surface degradation. Among several factors to determine MPs’ characteristics, MPs’ surface is degraded by external factors, such as ultraviolet (UV) light and surface chemical properties are modified^12^. However, due to the absence of standard samples that reflect the physical properties of MPs in the environment, assessments of their impact on human health have not been conducted. In other words, investigations using samples that reflect the physical properties of environmental MPs are essential for evaluating the actual impact of MPs on human health in real-world conditions. Despite the increasing concern regarding MPs in the environment and their potential health risks, little is known about whether the physicochemical properties of MPs, especially surface degradation, affect its cytotoxicity. In this context, although polyethylene (PE) degradation by UV light introduces carbonyl and hydroxy groups, cytotoxic response of degraded MPs and its precise mechanisms have rarely been reported. We have previously reported the creation of MPs samples with physicochemical properties similar to those of environmental MPs and demonstrated that these surface-degraded MPs exhibit cytotoxicity^13^.

In this study, we aimed to elucidate the biological mechanism of cytotoxicity exhibited by degraded PE. We evaluated the cytotoxic response of degraded PE on a phagocytic immune cell line by focusing on the type of cell death.

## Methods

### Cell culture

The mouse macrophage cell line RAW264.7 was purchased from the American Type Culture Collection (ATCC, Manassas, VA, USA). RAW264.7 cells were cultured in Dulbecco’s Modified Eagle Medium (high glucose) with L-glutamine and phenol red (Wako, Osaka, Japan) with 10% fetal bovine serum (FBS, Biosera, Nuaille, France) and 1% (*v*/*v*) penicillin–streptomycin-amphotericin B suspension (FUJIFILM Wako Pure Chemical, Osaka, Japan) and maintained at 37 °C at 5% CO_2_ and > 95% humidity. The human monocyte cell line THP-1 was purchased from ATCC. THP-1 cells were cultured in RPMI-1640 (FUJIFILM Wako Pure Chemical) with 10% FBS, 1% (*v*/*v*) penicillin–streptomycin–amphotericin B suspension, and 0.1% 2-mercaptoethanol (2-ME, Thermo Fisher Scientific) and maintained at 37 °C at 5% CO_2_ and > 95% humidity. Periodic tests for *Mycoplasma* were performed using commercially available kits (EZ-PCR™ Mycoplasma Detection Kit, Biological Industries, Beit-Haemek, Israel).

### PE degradation

In this study, we used flo-thene (Sumitomo Seika Chemicals Company, Osaka, Japan) as the PE particle sample. According to the manufacturer’s information, the medium particle size was 180 µm. We previously reported a degradation method for PE and confirmed its successful degradation^14^. In this study, PE degradation was conducted in accordance with a previous study^13,14^. Briefly, to degrade PE, we used a flat excimer ex mini (Hamamatsu Photonics K. K., Shizuoka, Japan), which irradiates UV light with a wavelength of 172 nm in the range of 86 × 40 mm. First, the PE sample was spread on the bottom of a Petri dish. It was placed approximately 10 mm away from the light source and irradiated with UV light for 30 min. The treated PE was collected from a sample bottle.

To confirm the PE degradation, attenuated total reflection infrared (ATR-IR) spectra were measured using an FT/IR-4700 (Jasco, Tokyo, Japan) with a TGS detector. A diamond ATR crystal (incident angle of 45°; approximately one reflection) set in a horizontal ATR accessory was used to measure the sample. All spectra were collected with 32 scans at 4 cm^−1^ resolution in the 4000–500 cm^−1^ range. First, the background spectrum without any sample on the ATR crystal (air) was measured and then we immediately performed sample measurement. The PE sample was then placed on the ATR crystal and pressed. IR spectra of the PE samples were recorded. The raw spectra are presented as the pATR (= − log I/I_0_) spectra, where the sample spectral intensity I was divided by the background spectral intensity I_0_ just before the sample measurement. To normalize the IR spectra, each value was normalized to the maximum value in each condition. Before further experiments, we tried several exposure times and analyzed the appropriate time duration of VUV exposure (Supplementary Fig. 1) considering the degree of degradation. To measure the degree of degradation, we utilized carbonyl index^15^. Briefly, carbonyl index was calculated by the ratio of the peak height of C=O (1715 cm^−1^) to that of C–H (1465 cm^−1^). In this study, we used degraded PE after 30 min VUV exposure with a carbonyl index ranging from 0.19 to 0.33, which is similar to the values of environmental samples^15,16^. We previously confirmed that surface of degraded PE was oxidized by inserting carbonyl groups and hydroxy groups using ATR-IR. In addition, X-ray photoelectron spectroscopy (XPS) was conducted to analyze the elements on the surface of materials, and XPS measurement showed that PE and degraded PE show no changes except for the introduction of carbonyl and hydroxy groups^13^. From the results of field emission scanning electron microscopy (FE-SEM), PE and degraded PE exhibit no alterations in terms of shapes^13^. In addition, it has been shown that average particle size of the PE and 1 h degraded PE samples were 231.4 µm and 231.9 µm, and particle less than 1 µm was also included in the PE and degraded PE samples by analysis with laser diffraction^13^.

### Annexin V assay

We performed annexin V (Bio Legend, San Diego, CA, USA) staining according to the manufacturer’s instructions. In brief, RAW264.7 and THP-1 cells were incubated with non-degraded/degraded PE. Carboxymethyl cellulose (CMC, 0.001%) was used as a dispersant in the medium. Staurosporine (200 nM; Cayman CHEMICAL) was used as a positive control. After 24 h of incubation, cells were harvested via trypsinization. Subsequently, 100 µL of cell suspension containing approximately 1 × 10^5^ cells were resuspended in 5 µL of FITC Annexin V at a concentration of 90 µg/mL plus 10 µL of propidium iodide (Sigma Aldrich, St. Louis, MO, USA) at a concentration of 1.25 mg/mL, followed by 15 min of incubation at room temperature protected from light. Then, cells were resuspended with 400 µL of binding buffer (2% FBS, 0.05% sodium azide (Wako) in phosphate-buffered saline (PBS). Stained cells were analyzed via flow cytometry using a MACSQuantX (Miltenyi Biotec, Bergisch Gladbach, Germany). The cells were gated based on side-scattered light (SSC) area, forward-scattered light (FSC) area, FSC height/FSC width, and SSC height/SSC width to eliminate doublet cells.

### Western blotting

RAW264.7 and THP-1 cells were treated with non-degraded or degraded PE at various concentrations for 1, 8, or 24 h. CMC (0.001%) was used as a dispersant in the medium. Proteins were extracted using RIPA (50 mM Tris–HCl; pH 7.5, 150 mM NaCl, 1% NP40, 0.1% sodium dodecyl sulfate (SDS), 0.5% sodium deoxycholate, and 1 mM EDTA) with protease and phosphatase inhibitor (Thermo Fisher Scientific, Waltham, MA, USA) and mixed with SDS sample buffer (6 ×) (0.375 M Tris [pH 6.8], 12% SDS, 60% glycerol, 0.06% bromophenol blue, and 600 mM dithiothreitol). The samples were boiled for 5 min, and the proteins were separated via SDS polyacrylamide gel electrophoresis (SDS-PAGE). The proteins were then electrotransferred onto a polyvinylidene difluoride membrane (Millipore, Bedford, MA, USA) and blocked with 4% (*w*/*v*) Block Ace (KAC Co., Ltd., Kyoto, Japan) in PBS containing 0.01% (*v*/*v*) Tween 20. The membranes were incubated with primary antibody (GAPDH: Cell Signaling Technology (CST, Danvers, MA, USA), 20,000 × , β-actin: Sigma-Aldrich, 50,000 × , Cleaved caspase3: CST, 1000 × , p-AMPK, CST, 1000 × , AMPK: CST, 5000 × , p-mTOR: CST, 1000 × , m-TOR: CST, 2000 × , LC3: Sigma-Aldrich, 1000 × , p62: CST, 1000 × , LAMP1: Santa Cruz Biotechnology (Dallas, TX, USA), 1000 ×) overnight at 4 °C. The membrane was then incubated with an anti-rabbit IgG-horseradish peroxidase-conjugated secondary antibody (1:2000; Cell Signaling Technology), and an anti-mouse IgG (Fab-specific)-peroxidase-conjugated secondary antibody (1:10,000; Sigma-Aldrich) for 1 h at room temperature. Protein bands were detected using ImmunoStar LD (Fujifilm) and visualized using an ImageQuant LAS 4000 mini biomolecular imager (GE Healthcare, Tokyo, Japan). Band intensity was measured using ImageJ software (1.54f.). β-actin and GAPDH were used as loading controls. All the full blots in the figures were shown in the supplementary figures. Since the blots were cut before incubation with primary antibodies, we have included all blots, including replicates.

### Cell viability assay

Cell viability was measured using MTT [3-(4,5-dimethylthiazol-2-yl)-2,5-diphenyl tetrazolium bromide] assay (Tokyo Chemical Industry, Tokyo, Japan) according to the manufacturer’s instructions. Cell viability was normalized to non-treated wells.

### Immunofluorescence

After treatment with non-degraded or degraded PE at 50 or 75 mg/mL for 24 h. CMC (0.001%) was used as a dispersant in the medium. Then, RAW264.7 cells were fixed with 4% paraformaldehyde for 15 min and permeabilized with 0.1% Triton X-100 for 5 min. After blocking with 1% BSA in PBS, cells were incubated overnight with anti LC3 antibodies (Sigma-Aldrich, 1000 ×) at 4 °C. Then, the antibodies were replaced 1% BSA in PBS containing Alexa Fluor™ 594 goat anti-rabbit (CST, dilution: 400 ×) as secondary antibody and DAPI (Sigma, dilution: 1000 ×) for 1 h. The cells were imaged using a CV8000 confocal microscope with a 60 × water immersion objective.

### Statistical analysis

Statistical analyses were performed using GraphPad Prism 9 for MacOS (version 9.5.0).

Results are presented as means ± SD. Statistical analyses were performed using one-way analysis of variance with Tukey’s multiple comparison test. (ns, not significant; *, *P* < 0.05; **, *P* < 0.01; ***, *P* < 0.001; ****, *P* < 0.0001).

## Results

### Degraded PE induced programmed cell death

**Fig. 1:**
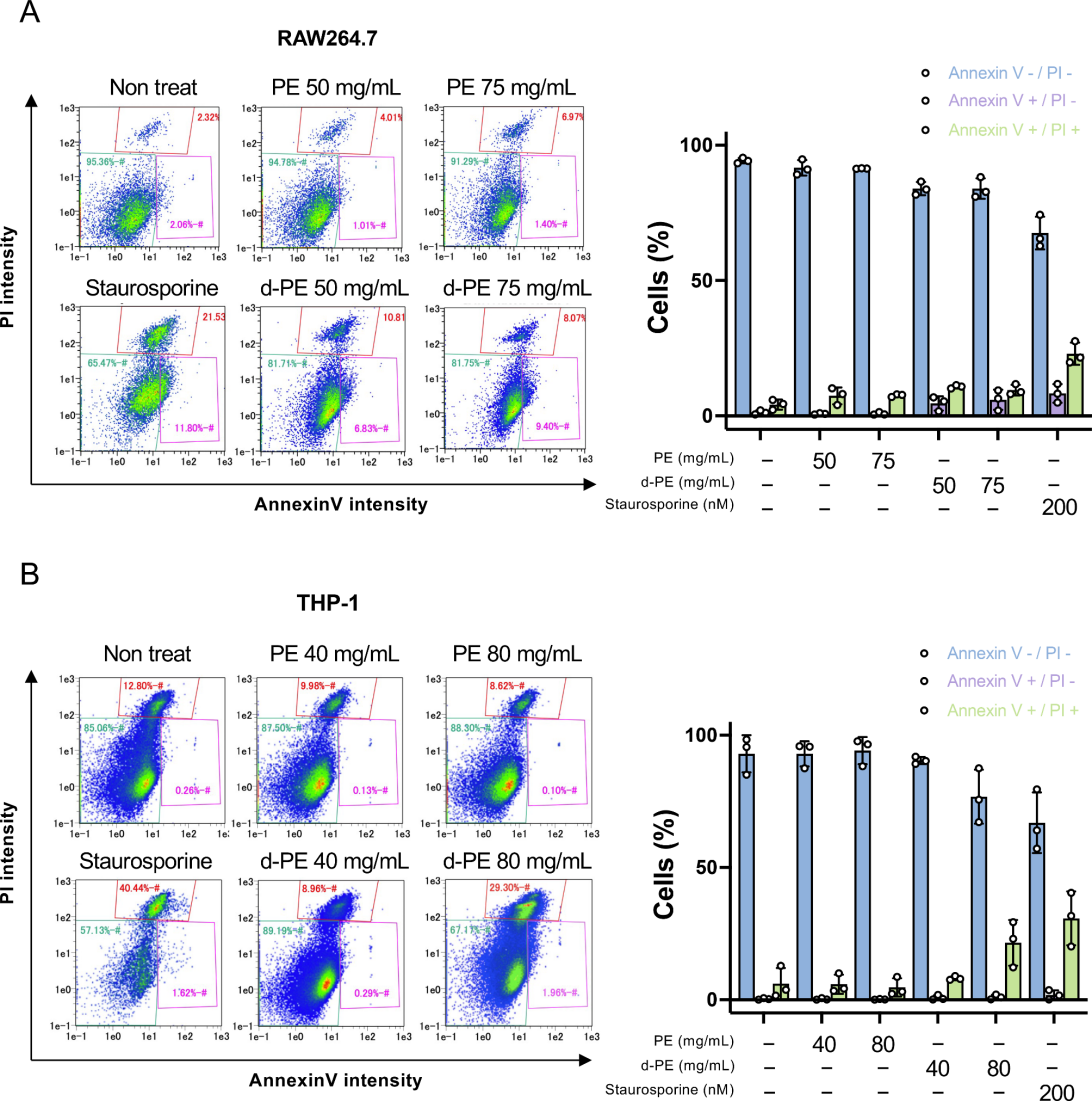
The effect of polyethylene microplastics on cell death of RAW264.7 and THP-1 cells. (**A**) RAW264.7 cells were seeded in 12-well plates at a density of 5.0 × 10^4^ cells per well and incubated for 24 h. Cell culture medium was then replaced by 100 μL of cell culture medium containing different concentrations of 50, 75 mg/mL of non-degraded PE or degraded PE samples and incubated for 24 h. Annexin V level and PI incorporation was determined by flow cytometry. Annexin V level and PI incorporation was determined by flow cytometry. Histogram at the right panel represent the cell population of PI-/Annexin V-, PI-/Annexin V + and PI + /Annexin V + in RAW264.7 cells. (**B**) THP-1 cells were seeded in 12-well plates at a density of 5.0 × 10^4^ cells per well and incubated for 24 h. Cell culture medium was then replaced by 100 μL of cell culture medium containing different concentrations of 40, 80 mg/mL of non-degraded PE or degraded PE samples and incubated for 24 h. Annexin V level and PI incorporation was determined by flow cytometry. Annexin V level and PI incorporation was determined by flow cytometry. Histogram at the right panel represent the cell population of PI-/Annexin V-, PI-/Annexin V + and PI + /Annexin V + in THP-1 cells. Data of each cell line were presented as the mean + S.D. of three independent experiments.

In this study, PE was selected as a model polymer type due to its high production and simple structure^17^. Additionally, since MPs have been detected in human blood^6^, two phagocytic immune cell lines—RAW264.7 (murine macrophage cells) and THP-1 (human monocyte cells)—were chosen as representative models for evaluating potential cytotoxic effects. To investigate whether non-degraded or degraded PE induced cell death, we assessed cell death using propidium iodide (PI) and annexin V staining in RAW264.7 cells and THP-1 cells. PI is a membrane-impermeable fluorescent probe for DNA and is used as an indicator of necrotic cell death. Annexin V is a fluorescent probe for surface phosphatidylserine (PS), which is localized to the cell surface in the early stages of programmed cell death. In RAW264.7 cells, which are murine macrophage cells, flow cytometric analysis showed that degraded PE exposure increased annexin V-positive cells, whereas almost no increase in cell death was observed in non-degraded PE-treated cells; the quantitative plot also showed that PI-/annexin V- cells were decreased and annexin V + cells were increased in degraded PE-treated cells (Fig. 1A). Similarly, in THP-1 cells, which are human monocyte cells, flow cytometric analysis showed that degraded PE exposure increased annexin V-positive cells, whereas almost no increase in cell death was observed in non-degraded PE-treated cells; the quantitative plot also showed that PI-/annexin V- cells were decreased and annexin V + cells were increased in degraded PE-treated cells (Fig. 1B). These results suggested that degraded PE induced programmed cell death.

### Degraded PE induced cell death other than apoptosis

**Fig. 2:**
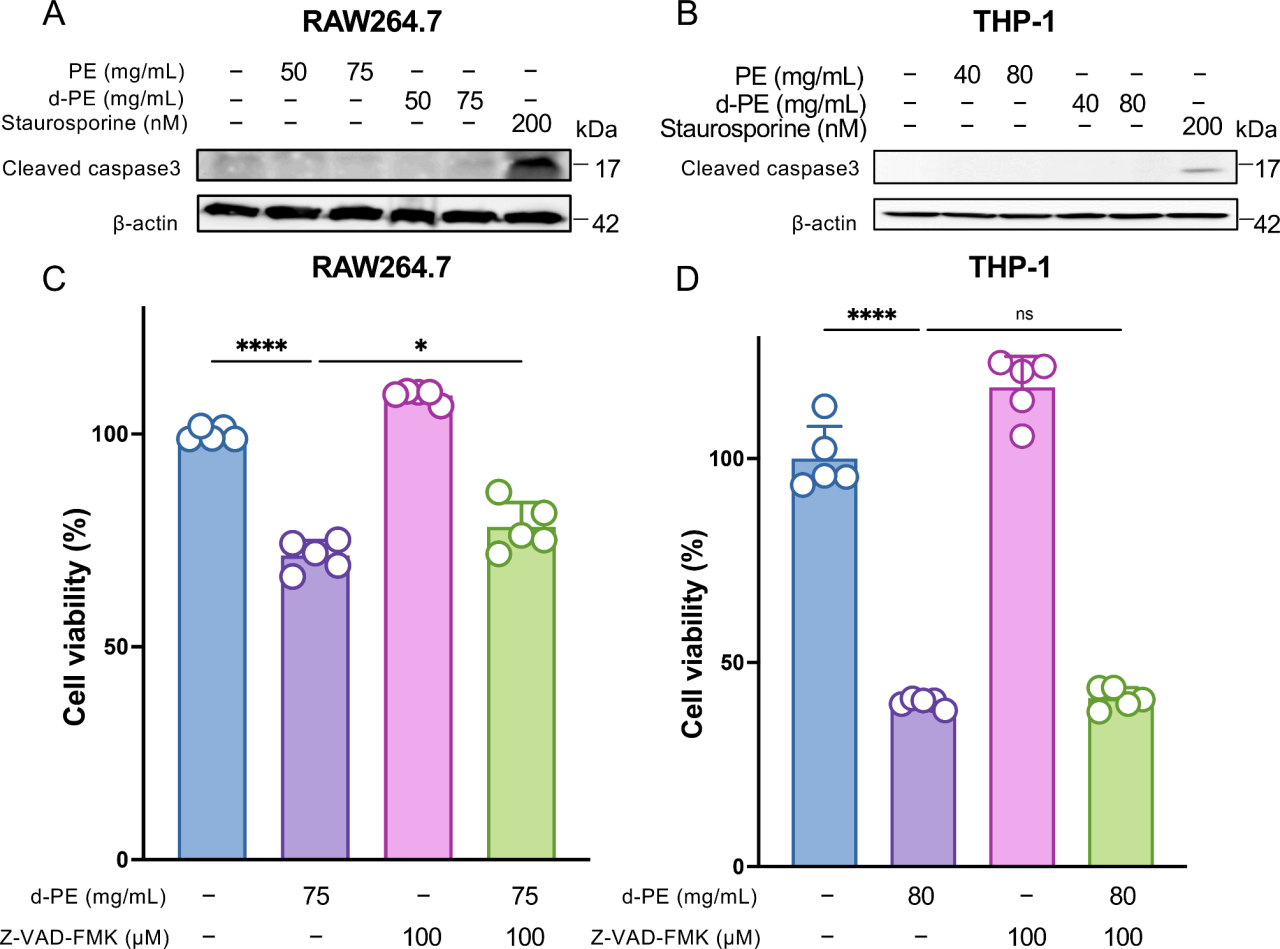
Degraded PE-induced cell death and relation of apoptosis on RAW264.7 and THP-1 cells. (**A**) RAW264.7 cells were seeded in 6-well plates at a density of 2.0 × 10^5^ cells per well and incubated for 24 h. Cell culture medium was then replaced by cell culture medium containing 50, 75 mg/mL of non-degraded PE or degraded PE and incubated for 24 h. After incubation, whole cell extracts were analyzed using electrophoresis and immunoblotting with the indicated antibodies. β-actin was used as loading control. (**B**) THP-1 cells were seeded in 6-well plates at a density of 2.0 × 10^5^ cells per well and incubated for 24 h. Cell culture medium was then replaced by cell culture medium containing 40, 80 mg/mL of non-degraded PE or degraded PE samples and incubated for 24 h. After incubation, whole cell extracts were analyzed using electrophoresis and immunoblotting with the indicated antibodies. β-actin was used as loading control. (**C**) RAW264.7 cells were seeded at 1 × 10^4^ cells per well in 96 well plate and treated with 100 μM z-VAD-fmk for 24 h, and then treated with degraded PE either alone or comibination with with 100 μM z-VAD-fmk for another 24 h. Cell viability was determined by MTT assay. The bar graph represent means ± SD (n = 5). The experiment was repeated twice with similar results. (**D**) THP-1 cells were seeded at 1 × 10^4^ in 96 well plate and pretreated with 100 μM z-VAD-fmk for 24 h, and then treated with degraded PE either alone or comibination with with 100 μM z-VAD-fmk for another 24 h. Cell viability was determined by MTT assay. The bar graph represent means ± SD (n = 5). The experiment was repeated twice with similar results. Note that significance was assessed in (**C**) and (**D**) using one way ANOVA followed by Tukey’s method. n.s., not significant; *, *P* < 0.05; ****, *P* < 0.0001.

To further explore the molecular mechanism of degraded PE-induced cell death, we assessed cleaved caspase 3, which is known as a commonly used indicator of apoptosis^18^. Western blotting analysis showed that non-degraded and degraded PE did not activate caspase 3 in both RAW264.7 and THP-1 cells (Fig. 2A,B). In addition, to confirm that degraded PE-induced cell death was not caspase-dependent, cells were treated with either degraded PE alone or with the pan-caspase inhibitor Z-VAD-FMK, which inhibits apoptosis^19^. In RAW264.7 cells, Z-VAD-FMK treatment slightly suppressed the cytotoxicity of degraded PE (Fig. 2C). In THP-1 cells, Z-VAD-FMK did not suppress the cytotoxicity of the degraded PE (Fig. 2D). These results suggest that degraded PE might induce programmed cell death other than caspase-driven apoptosis.

### Degraded PE promoted autophagosome formation

**Fig. 3:**
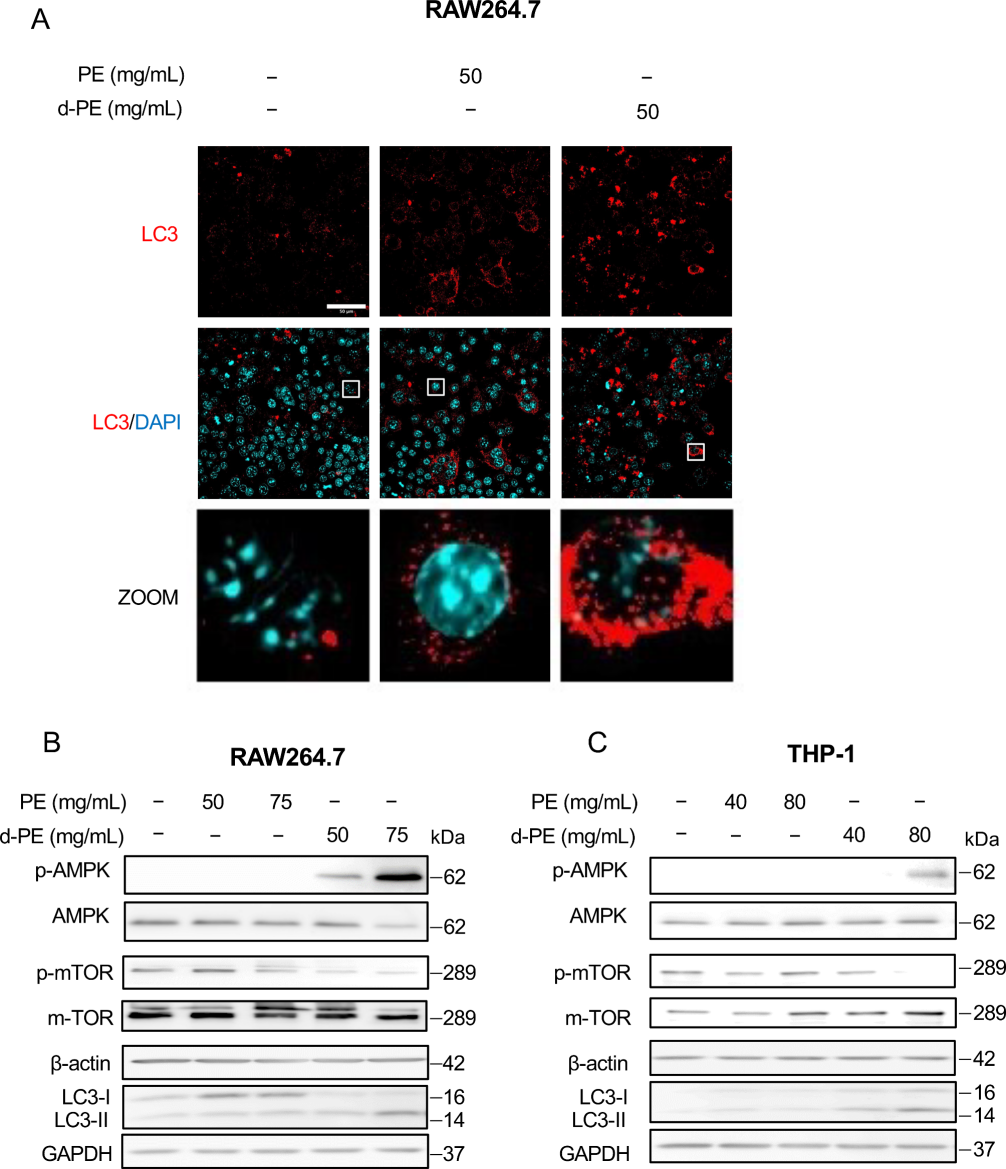
Autophagosome accumulation caused by degraded PE. (**A**) RAW264.7 cells were seeded at 1 × 10^5^ cells per well in 96 plate and then incubated for 24 h. After incubation, cells were treated with non-degraded PE and degraded PE and incubated for 24 h. Then, cells were fixed and permeabilized. After blocking, cells were incubated overnight with anti-LC3 antibody overnight at 4 °C. Then, cells were incubated with secondary antibody with DAPI for 1 h. Scale bar: 50 µm. (**B**) RAW264.7 cells were seeded at 2 × 10^5^ cells per well in 6 well plate and treated by non-degraded PE or degraded PE samples at the indicated concentrations and incubated for 24 h. After incubation, whole cell extracts were analyzed using electrophoresis and immunoblotting with the indicated antibodies. β-actin and GAPDH were used as loading control. The experiment was repeated twice with similar results. (**C**) THP-1 cells were seeded at 2** × **10^5^ cells per well in 6 well plate and treated by non-degraded PE or degraded PE samples at the indicated concentrations and incubated for 24 h. After incubation, whole cell extracts were analyzed using electrophoresis and immunoblotting with the indicated antibodies. β-actin and GAPDH were used as loading control. The experiment was repeated twice with similar results.

As degraded PE promoted cell death that was not accompanied with caspase-3 activation, we focused on how cells responded to d-PE-induced cellular stress. Autophagy is one of the major cellular responses that contributes to cell survival by degrading and recycling damaged cell compartments, including cellular organelles^20^. Additionally, dysregulation of autophagy leads to cell death^21^. Therefore, we first evaluated autophagosome formation by staining for LC3, which is localized to autophagosome membranes^22^. Immunofluorescence analysis demonstrated accumulation of autophagosomes in degraded PE-exposed RAW264.7 cells (Fig. 3A). We examined the expression of autophagy-related molecules. As a result, in degraded PE-treated cells, phosphorylation of AMPK and LC3-II/LC3-I ratio was increased, while m-TOR phosphorylation was decreased in both RAW264.7 and THP-1 cells (Fig. 3B,C). Collectively, these results suggest that degraded PE activated autophagosome formation in RAW264.7 cells.

### Degraded PE disrupted autophagy flux

**Fig. 4:**
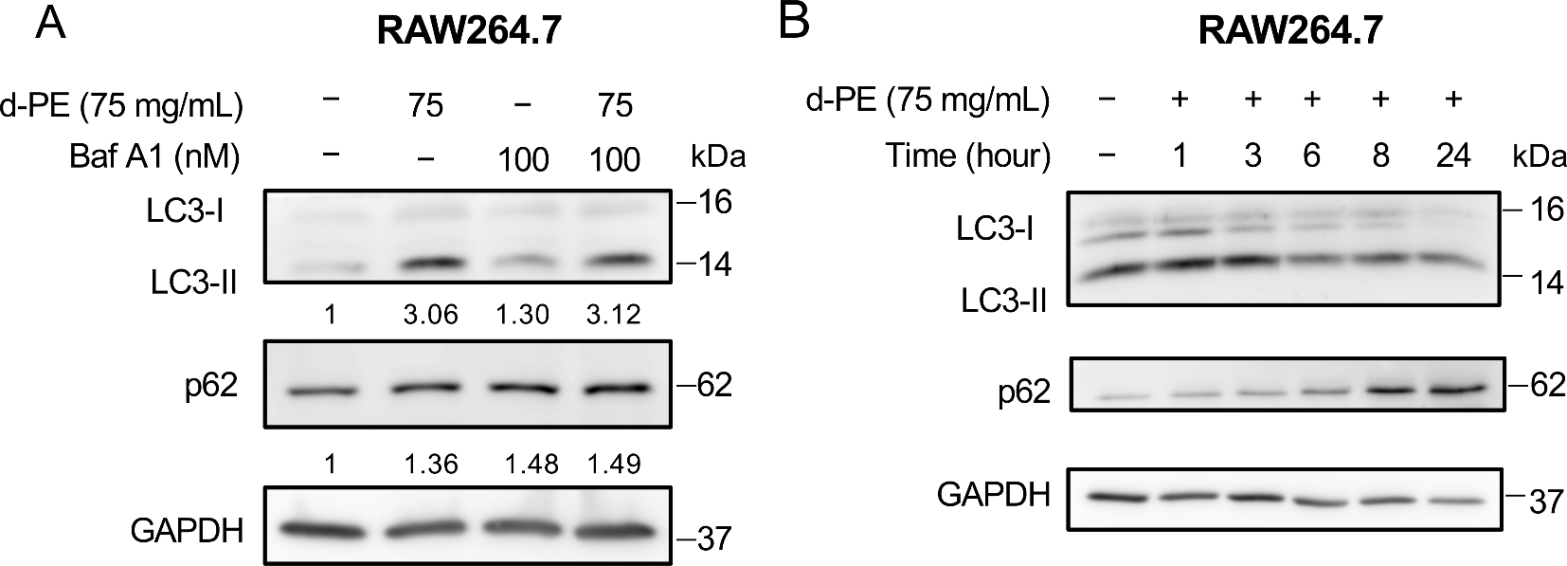
Autophagy disruption by degrade PE treatment. (**A**) RAW264.7 cells were seeded at 1 × 10^5^ cells per well in 6 well plate and treated with 50 mg/mL degraded PE either alone or combination with 100 nM of Bafilomycin A1 and incubated for 24 h. After incubation, whole cell extracts were analyzed using electrophoresis and immunoblotting with the indicated antibodies. GAPDH was used as loading control. The experiment was repeated twice with similar results. (**B**) RAW264.7 cells were seeded at 1 × 10^5^ cells per well in 6 well plate and treated with 75 mg/mL degraded PE for the indicated time. After incubation, whole cell extracts were analyzed using electrophoresis and immunoblotting with the indicated antibodies. GAPDH was used as loading control. The experiment was repeated twice with similar results.

As we observed autophagosome accumulation in degraded PE-treated cells, we next assessed whether autophagy was inhibited or promoted by exposure to degraded PE. To interpret the autophagy flux, we conducted an autophagy flux assay^23^. RAW264. 7 cells were exposed to either degraded PE, an autophagy inhibitor, Bafilomycin A1 alone, or a combination of degraded PE and Bafilomycin A1. As shown in Fig. 4A, although exposure to degraded PE increased the ratio of LC3-II/LC3-I, the combination of degraded PE and autophagy inhibitor did not alter the ratio of LC3-II/LC3-I, indicating that degraded PE-exposed RAW264.7 cells were autophagy-dysregulated, and the degradation process was blocked^23^. We also measured p62 (SQSTM1/sequestosome 1) expression as an indicator of autophagic flux^23^. Immunoblotting showed that p62 accumulated upon exposure to degraded PE and was not altered by combined treatment with degraded PE and an autophagy inhibitor (Fig. 4A). These data suggest that autophagy was suppressed by PE degradation. To further confirm the time kinetics of the ratio of LC3-II/LC3-I and p62 accumulation, RAW264.7 cells were treated with degraded PE for 1, 3, 6, 8, and 24 h, and LC3-I, LC3-II, and p62 were detected via immunoblotting. The results showed that both the LC3-II/LC3-I ratio and p62 accumulation increased in a time-dependent manner (Fig. 4B).

### Degraded PE reduced acidic lysosomes

**Fig. 5:**
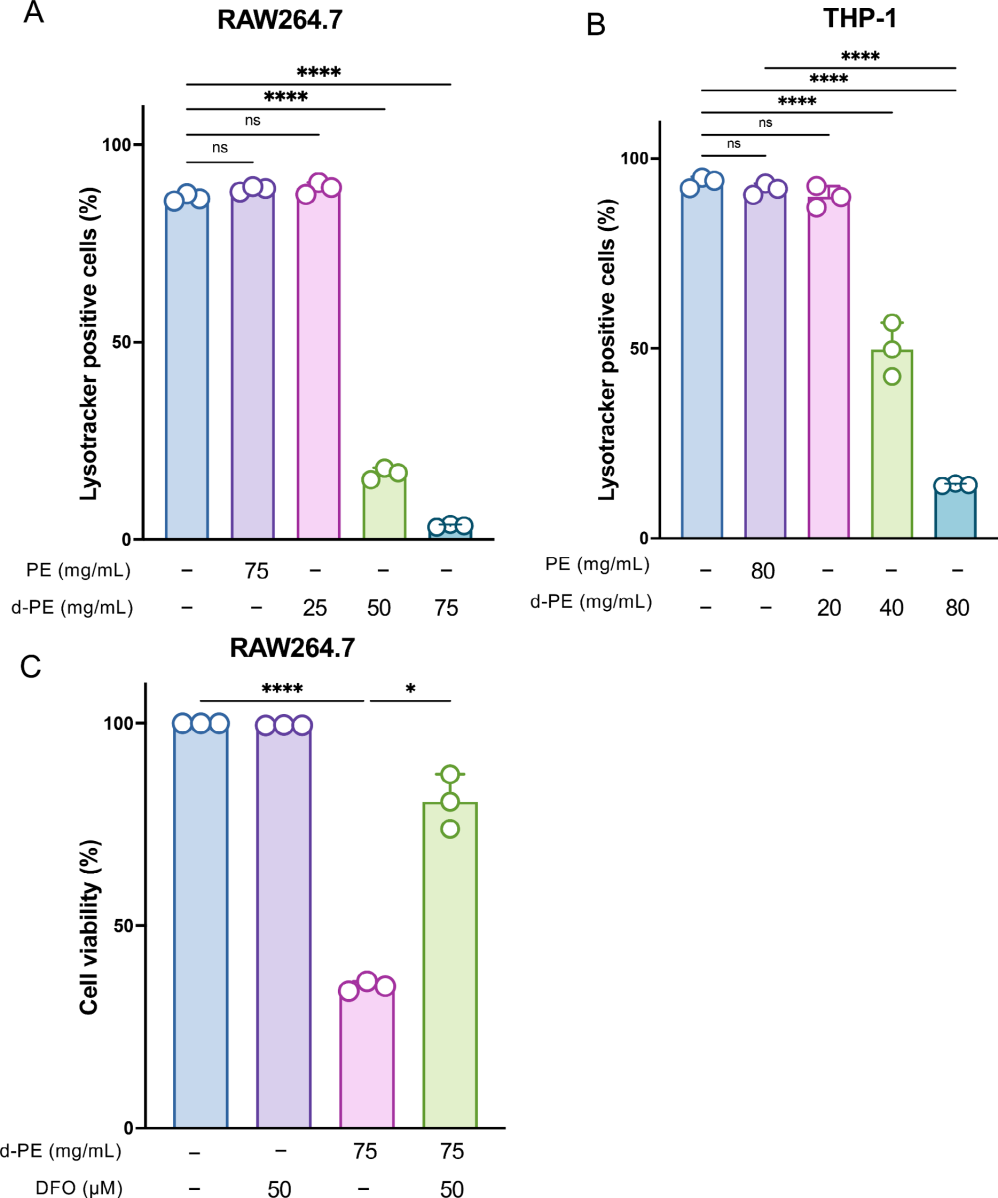
Degraded PE caused lysosome dysregulation in both RAW264.7 cells and THP-1 cells. (**A**) RAW264.7 cells were seeded at 1 × 10^5^ cells per well in 6 well plate and incubated for 24 h. Then, cells were treated with non-degraded PE or degraded PE at the indicated concentration for 16 h. After incubation, cells were stained with lysotracker Green and analyzed using flowcytometry. The experiment was repeated twice with similar results. (**B**) THP-1 cells were seeded at 1 × 10^5^ cells per well in 6 well plate and incubated for 24 h. Then, cells were treated with non-degraded PE or degraded PE at the indicated concentration for 16 h. After incubation, cells were stained with lysotracker Green and analyzed using flowcytometry. The experiment was repeated twice with similar results. (**C**) RAW264.7 cells were seeded at 1 × 10^4^ cells per well in 96 plate and then incubated for 24 h. After incubation, cells were treated with degraded PE either alone or combination with DFO (50 µM) for 24 h. Cell viability was determined by MTT assay. Data were presented as the mean + S.D. of three independent experiments. Note that significance was assessed using one way ANOVA followed by Tukey’s method. n.s., not significant; ***, *P* < 0.001; ****, *P* < 0.0001.

To clarify the mechanisms underlying the dysregulated autophagy, we investigated the functions of lysosomes. Functional lysosomes are under acidic conditions at approximately pH 5, and Lysotracker probes have been used for the detection of acidic lysosomes^24^. RAW264.7 and THP-1 cells were treated with either non-degraded PE or degraded PE, and the fluorescence intensity of Lysotracker was evaluated. As shown in Fig. 5A,B, PE degradation significantly decreased the number of Lysotracker-positive cells, whereas exposure to non-degraded PE did not alter Lysotracker-positive cells. These data suggest that PE degradation promotes lysosomal damage. To assess the effect of degraded PE-mediated lysosomal damage on cell death, we focused on the relationship between lysosomal components and cell death. As lysosomes act as storage sites for iron and its release into the cytoplasm can cause severe damage to cells^25^, we analyzed the effect of iron chelation on degraded PE-mediated cell death using deferoxamine (DFO), an iron chelator, due to its high affinity for iron^24^. In RAW264.7 cells, co-treatment with degraded PE and DFO rescued the cell toxicity caused by degraded PE treatment (Fig. 5C), indicating that iron in the cell caused degraded PE-mediated cell death. This suggests that the cytotoxic effects of degraded PE are mediated through iron-dependent mechanisms, specifically ferroptosis. To confirm this ferroptotic event, we measured lipid peroxidation, as lipid peroxidation is accumulated in ferroptosis^26^, and examined whether this event is dependent on the degradation rate on the surface of PE. Our results showed that lipid peroxidation levels correlated with the degradation rate, as determined by the carbonyl index (Supplementary Figs. 1 and 2A). Additionally, to further verify the involvement of ferroptosis, we quantified the expression of the gene SLC40A1, which is known to be upregulated during ferroptosis^27^. PCR analysis revealed that degraded PE (d-PE) induces the expression of SLC40A1 (Supplementary Fig. 2B). These findings collectively suggest that degraded PE induces lysosome-deregulated cell death, potentially related to ferroptosis, in RAW264.7 cells.

## Discussion

Plastics are extensively utilized in various products owing to their distinctive properties, including strength, durability, and cost-effective manufacturability^28^. Although, there is a growing focus on plastic accumulation in the environment and global concern regarding MPs, which are tiny plastic fragments, has gained significant traction^29^, little is known about how MPs in the environment affect human health. Considering the complex physicochemical properties of MPs in terms of size, shape, polymer type, and surface degradation, it is necessary to reflect these physicochemical factors (s) in the potential impact of MPs on human health. In this study, we focused on the surface degradation of MPs and attempted to evaluate the cell toxicity mechanisms. Mechanistically, surface-degraded MPs induce nonapoptotic cell death and lysosomal dysfunction.

The present study demonstrated that degraded PE promoted lysosomal damage and cell death, whereas non-degraded PE did not exert the same effect. As the density of PE is lower than that of water^30^, even when cells are exposed to a mixture of PE and carboxymethyl cellulose (CMC) as a dispersant in the medium, the likelihood of interactions between cells and PE remains minimal. In this context, the potential factors underlying the selective cell toxicity of degraded PE in two immune cell types, THP-1 and RAW264.7, could include: (1) alterations in the protein coating composition (protein corona), (2) release of monocarboxylic acid or dicarboxylic acid from the PE surface, and (3) cellular harm or uptake upon contact with degraded PE. Our earlier investigations revealed that both PE and 1-h degraded PE samples had an average particle size of approximately 231.4 µm and 231.9 µm, respectively, with particles smaller than 1 µm present in both PE and degraded PE samples^13^. Further investigation is required to evaluate the potential cellular uptake of degraded PE and its contribution to lysosomal damage by utilizing fluorescent labeled particles which could potentially be achieved with Nile Red^31^.

Although the maximum concentration of PE in the human blood is 7.1 µg/mL^6^, the concentration of PE in this study (40–80 mg/mL) was much higher than realistic environment concentration. Therefore, future experiments should include a broader range of concentrations, especially lower ones, in both cell death analysis and lysosome functional assays. In addition, given the earlier mention of the limited direct interactions between cells and PE due to its density, additional experiments using a rotary cell culture system with environment relevant concentrations are warranted. This system facilitates convenient interactions between the cells and PE at environmentally relevant concentrations.

In addition to PE, which encompasses both low-density polyethylene (LDPE) and high-density polyethylene (HDPE), other polymers such as polypropylene (PP), polystyrene (PS), polyethylene terephthalate (PET), and polyvinyl chloride (PVC) merit consideration due to their widespread production and substantial contribution to environmental waste^17^. Each of these polymers exhibits distinct physical and chemical properties, influencing their persistence and interaction with biological systems. Understanding their environmental fate and potential impacts on organisms is essential for comprehensive environmental risk assessment strategies.

Degraded PE-induced cytotoxicity was rescued by DFO treatment (Fig. 5C) and degraded PE induced lipid peroxidation (Supplementary Fig. 2A), suggesting a potential association between intracellular ferrous ions and cell death in degraded PE-treated cells because DFO treatment suppresses ferroptosis. Ferroptosis, a novel form of cell death discovered in 2012, is driven by the iron-dependent accumulation of lipid peroxidation^26^. Regarding particle-induced cytotoxicity and ferroptosis, it has been reported that silver nanoparticles induced ferroptosis in macrophages^32^, and zinc oxide nanoparticles induces ferroptosis in neuronal cell^33^. Considering these findings, we hypothesize a potential link between degraded PE and ferroptosis, which may contribute to ferroptosis-related diseases such as neurodegeneration and lung fibrosis^34^. Consequently, additional investigations are needed to ascertain whether degraded PE-induced cell death is linked to ferroptosis. For instance, the exploration of intracellular ferrous ions and the expression dynamics of ferroptosis-related genes is warranted.

To further develop this research and bridge the gap towards evaluating the health impacts of microplastics on humans, in vivo studies are essential considering the potential exposure routes, such as oral, inhalation, and dermal exposure^35^. While this study focused on MPs, future research should also assess the effects of nanoplastics (NPs), which are smaller than 1 µm^36^. By evaluating whether ferroptosis, as observed in this study, could also be induced by NPs, and by investigating the effects of surface-degraded NPs, we can gain insights into the size-dependent differences in their biological impacts.

In the present study, we showed that the surface degradation of PE caused cell toxicity. Given the fact that MPs in the environment have a variety of physicochemical properties, such as size, shape, polymer type, and surface degradation^37–39^, evaluation of MPs on biological response should consider these physicochemical properties. Our findings highlight the importance of considering the degradation state of MPs during cellular toxicity.

## Conclusions

In this study, we utilized degraded PE through UV irradiation and demonstrated that degraded PE exposure results in cell death attributed to lysosomal dysfunction. These findings provide novel insights into the effects of surface-degraded MPs on biological responses.

## Supplementary Information

Supplementary Information.

## Data Availability

All data analyzed within this study are included either in the manuscript or supplementary information files.

## Abbreviations

ATR-IR: Attenuated total reflection infrared
CMC: Carboxymethyl cellulose
DFO: Deferoxamine
MPs: Microplastics
PE: Polyethylene
PI: Propidium iodide
UV: Ultraviolet
