# Supplementary material for: Treatment of polyethylene microplastics degraded by ultraviolet light irradiation causes lysosome-deregulated cell death
Source: Sci Rep. 2024 Oct 14;14:24008. doi: 10.1038/s41598-024-74800-y (PMC11473831; doi:10.1038/s41598-024-74800-y)

Material and Method

Lipid peroxidation

RAW264.7 cells were seeded at $1x10^5$ cells per well in 12 well plate and treated with non-degraded PE and degraded PE with different CI at the concentration of 25 mg/mL for 24 h. After incubation, cells were stained with BODIPY™ 581/591 C11. Stained cells were analyzed via flow cytometry using a MACSQuantX (Miltenyi Biotec, Bergisch Gladbach, Germany). The cells were gated based on side-scattered light (SSC) area, forward-scattered light (FSC) area, FSC height/FSC width, and SSC height/SSC width to eliminate doublet cells.

Real time RT-PCR

RAW264.7 cells were seeded at $1x10^5$ cells per well in 12 well plate and treated with non-degraded PE and degraded PE at the concentration of 25 mg/mL for 24 h. Total RNA was extracted by using a FastGene RNA Kit (Nippon Genetics, Tokyo, Japan) and reverse-transcribed into cDNA by using a High-Capacity cDNA Reverse Transcription Kit (Thermo Fisher Scientific, Waltham, MA, USA). A PCR mixture was prepared containing the above cDNA as a template and primers for the genes, SLC40A1 (forward, 5'-GGCACTTTGCAGTGTCTGTG -3’ and reverse, 5'-GTCACCAATGATGGCTCCCA-3'), and actin (forward, 5'-ACGGCCAGGTCATCACTATTG-3' and reverse, 5'-CAAGAAGGAAGGCTGGAAAAGA-3') (Eurofins Genomics, Tokyo, Japan), as well as GeneAce SYBR qPCR Mix α Low ROX (Nippon Gene, Tokyo, Japan). RT-PCR was performed by using a CFX-384 Real-Time PCR Detection System (BioRad Laboratories, Hercules, CA, USA). The expression level of each gene was normalized to that of β-actin.

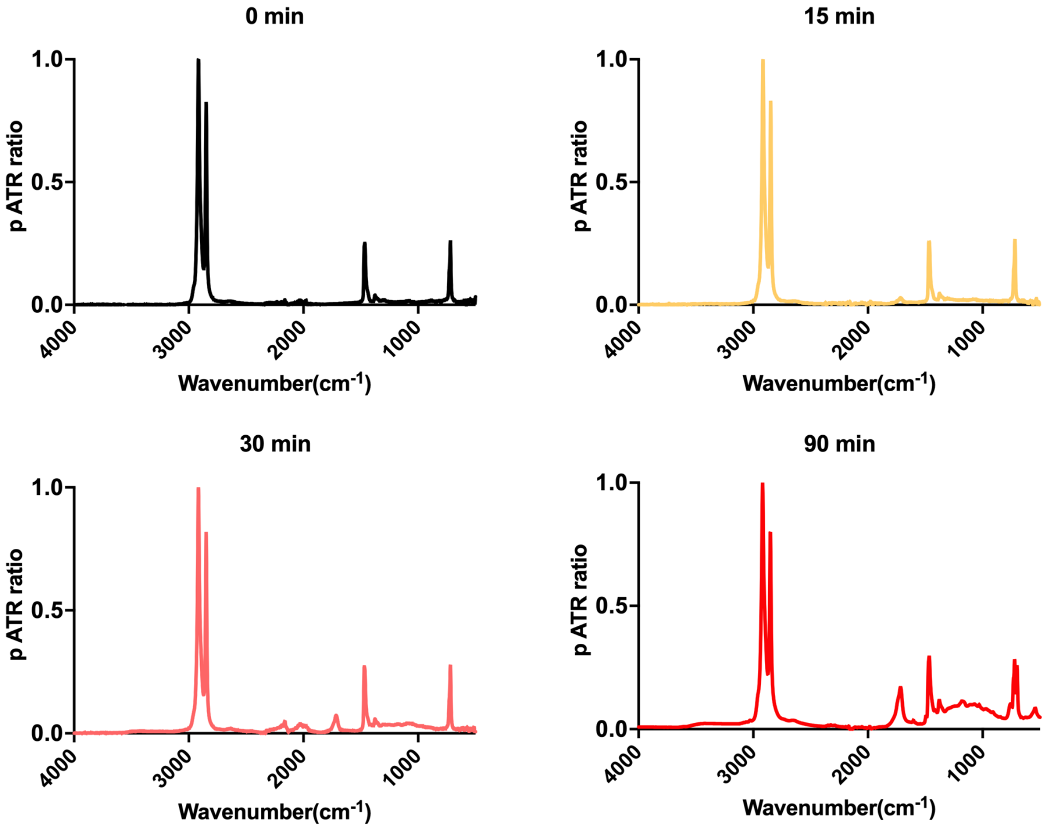

Supplementary figure 1. Surface properties of degraded PE by ATR-IR. Raw ATR-IR spectra of PE samples after several times of VUV exposure (0, 15, 30, and 90 min).

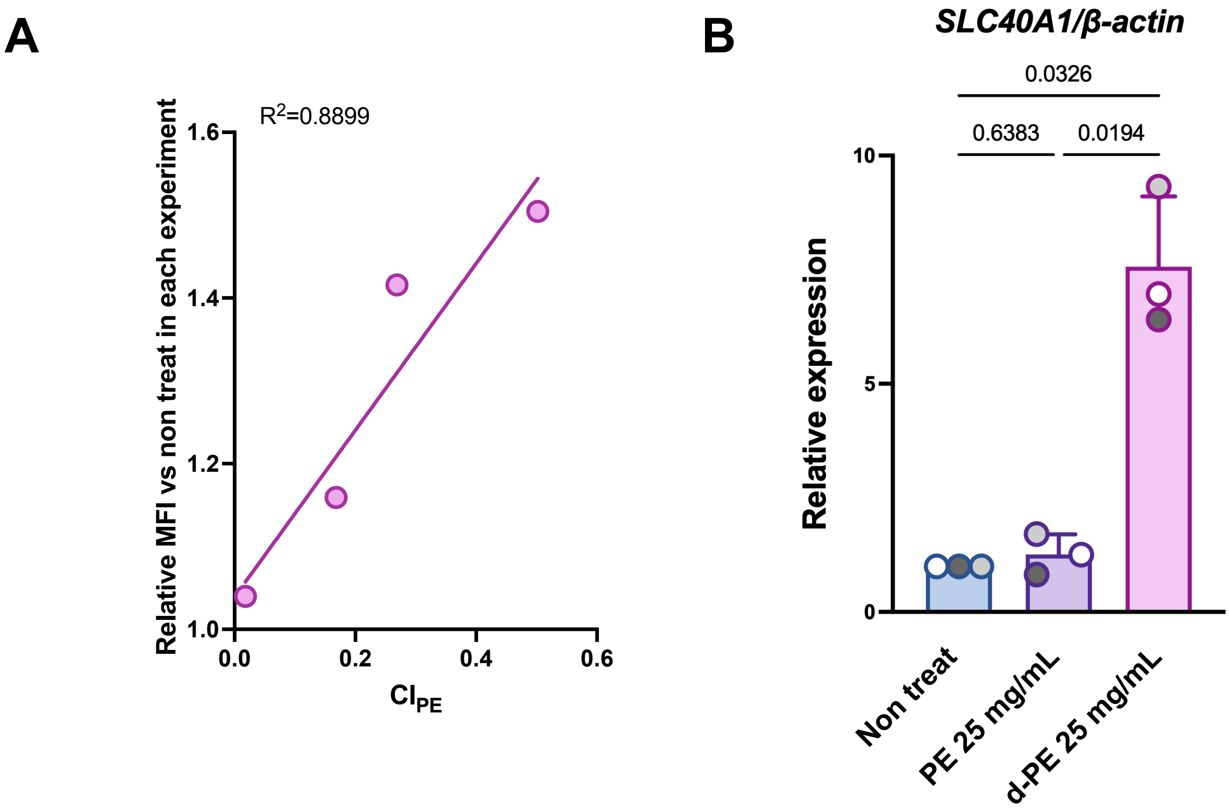

Supplementary figure 2. Surface degradation of PE and lysosomal damage-related events.
(A) RAW264.7 cells were seeded at $1x10^5$ cells per well in 12 well plate and treated with of 25 mg/mL non-degraded PE and degraded PE with different carbonyl index (CI) at the concentration for 24 h. After incubation, cells were stained with BODIPY™ 581/591 C11 and analyzed using flowcytometry. CI was calculated by using the spectra of supplementary figure 1. **(B)** RAW264.7 cells were seeded at $1x10^5$ cells per well in 12 well plate and treated with non-degraded PE and degraded PE at the concentration of 25 mg/mL for 24 h and then total RNA was extracted and analyzed for Real time RT-PCR. Data were presented as the mean +S.D. of three independent experiments. Significance was assessed using one way ANOVA followed by Tukey's method.

Supplementary figure 2. We present original images of immunoblots in all figures and repeated experiments. The blot was cut before hybridization with antibodies.

Fig. 2A

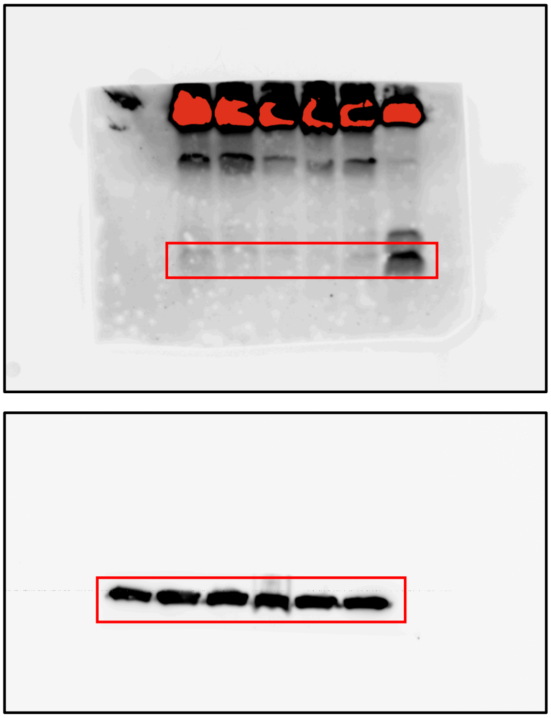

Cleaved caspase3

β-actin

Fig. 2A repeated

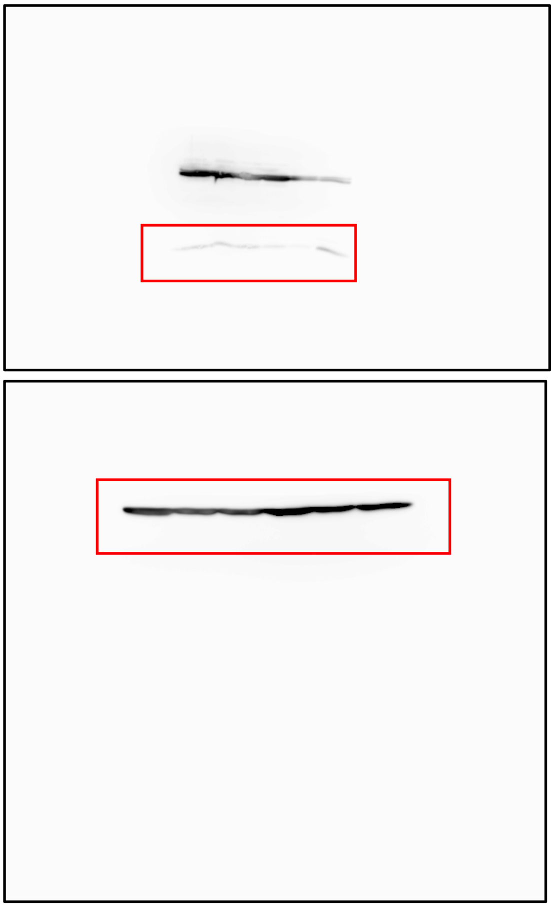

Cleaved caspase-3

β-actin

Fig. 2B

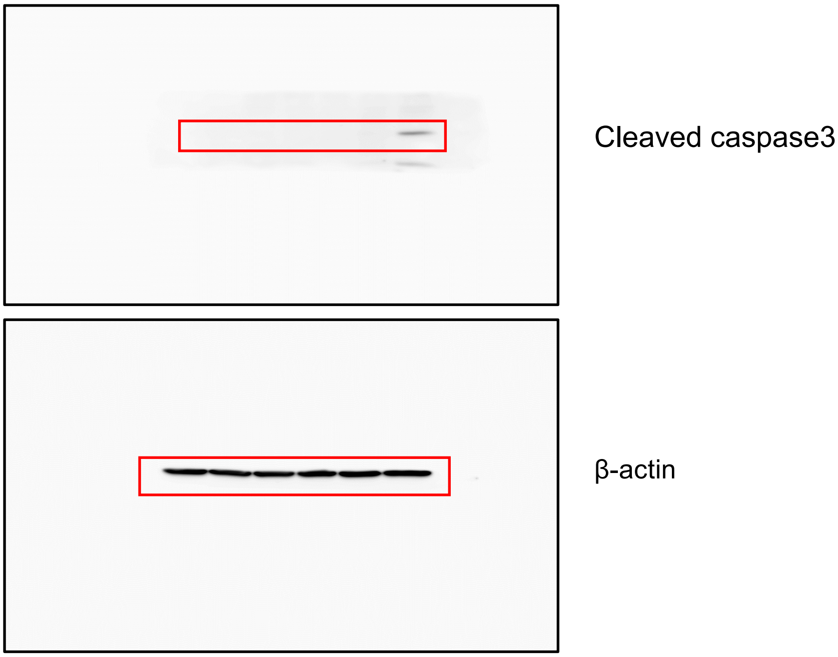

Fig. 2B repeated

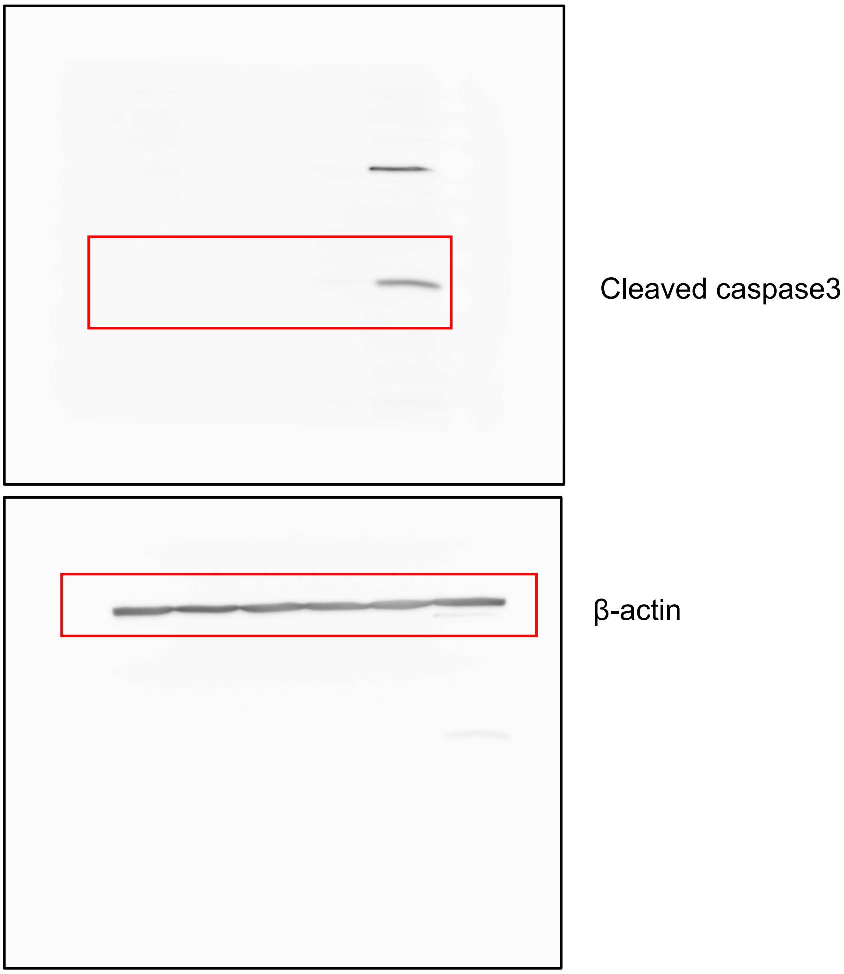

Fig. 3B

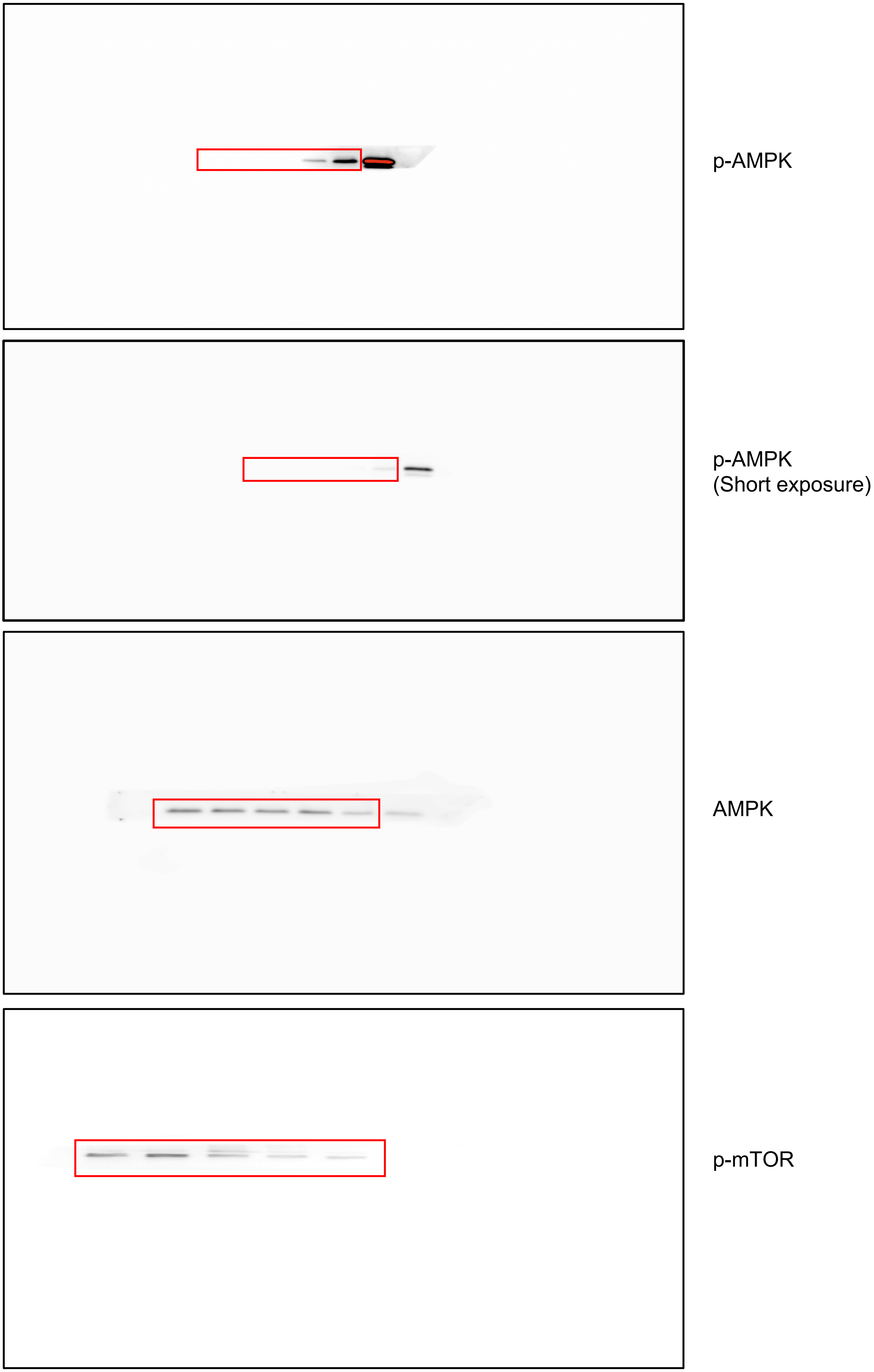

Fig. 3B (continued)

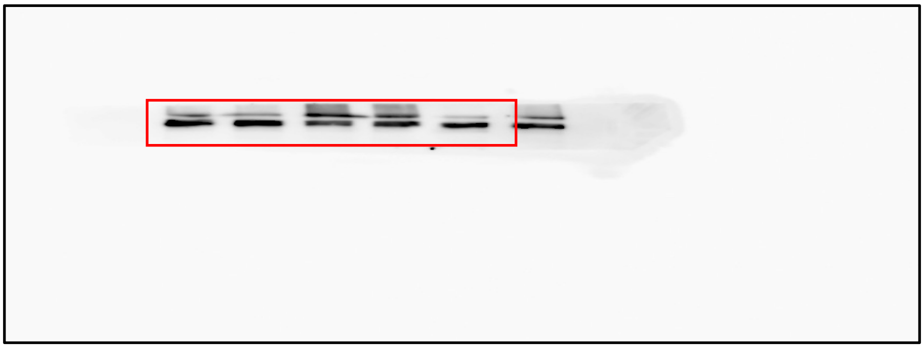

mTOR

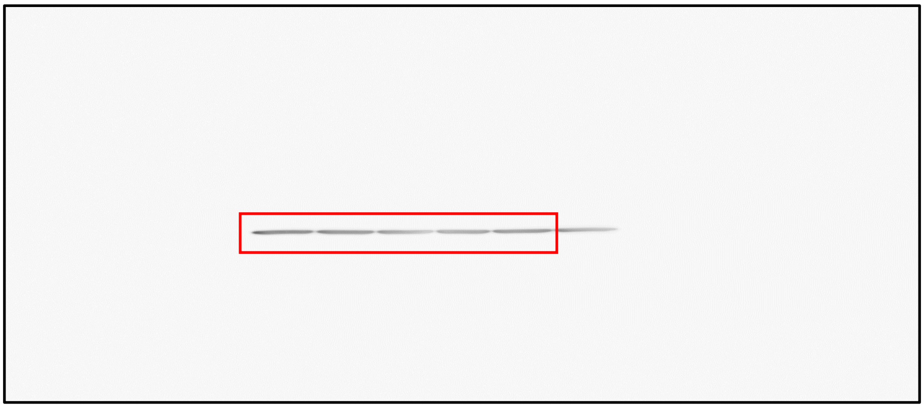

β-actin

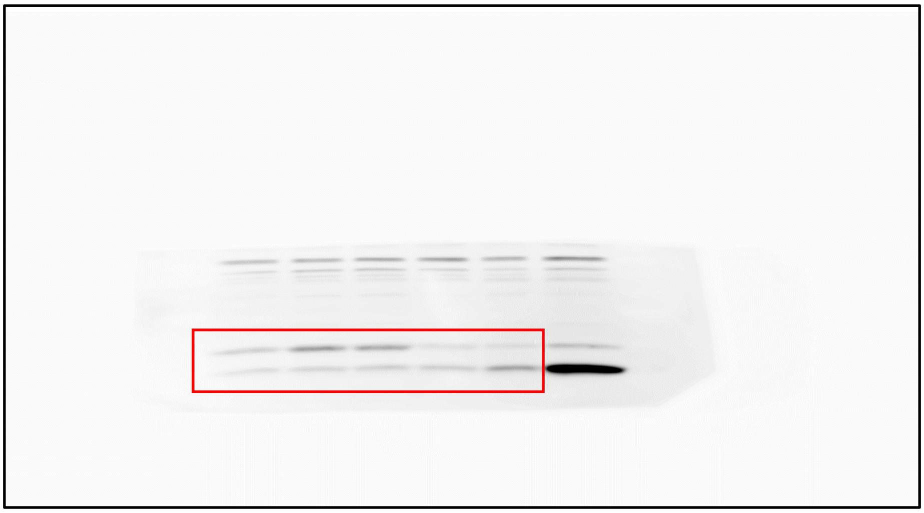

LC3

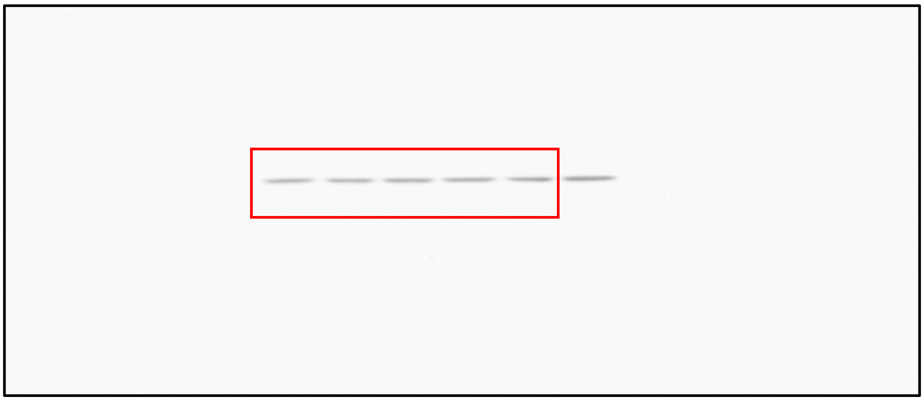

GAPDH

Fig. 3B repeated

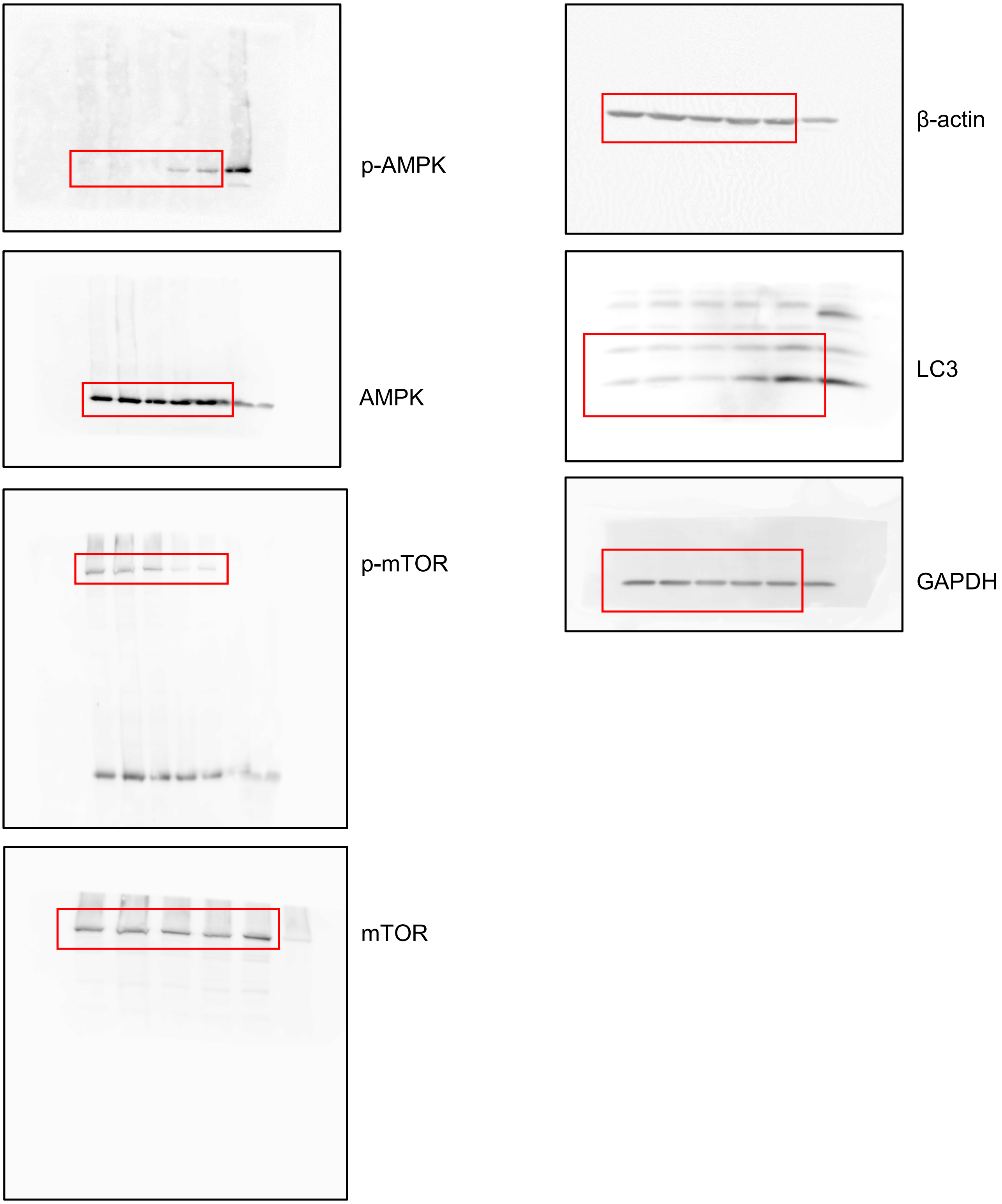

Fig. 3C

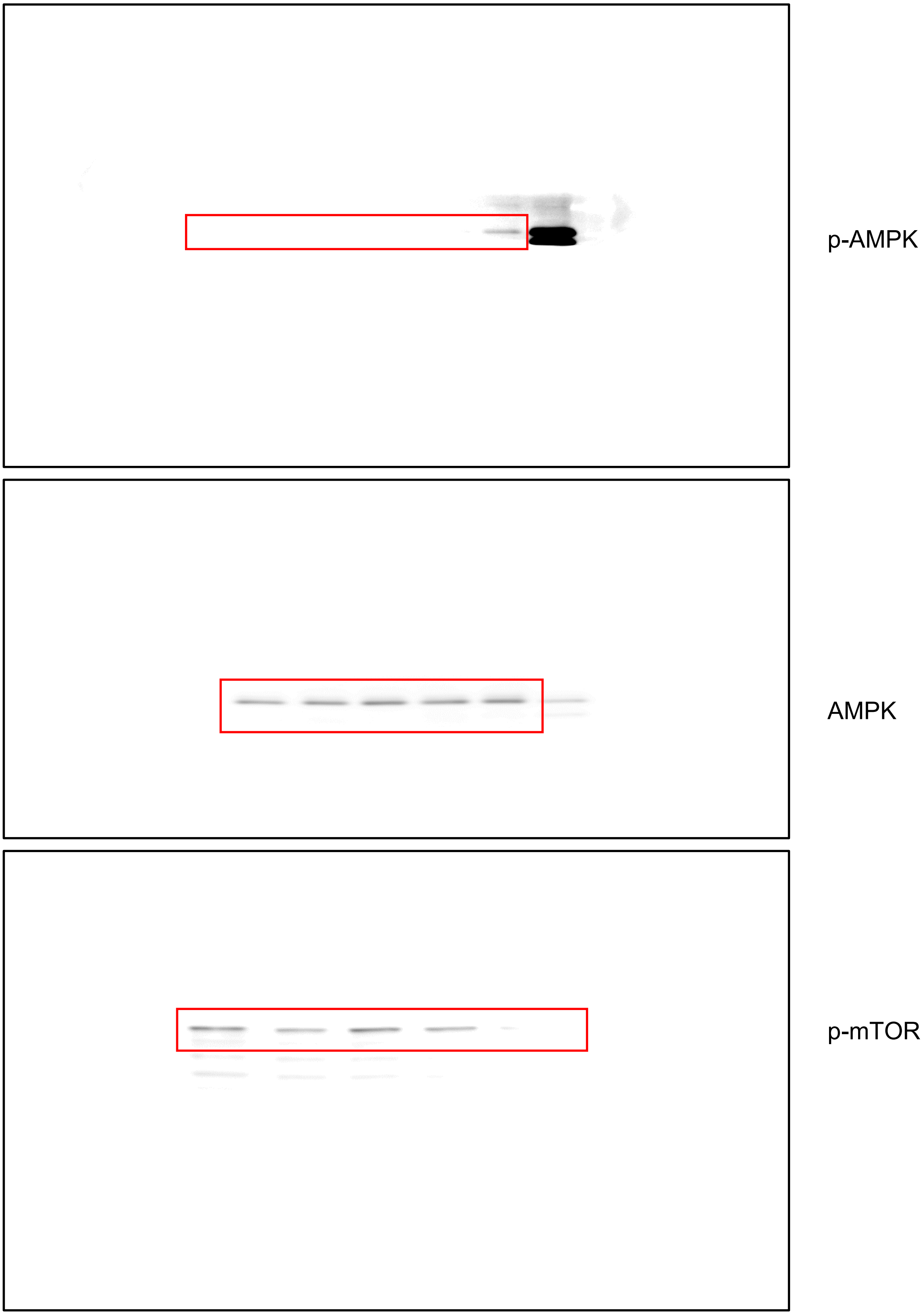

Fig. 3C (continued)

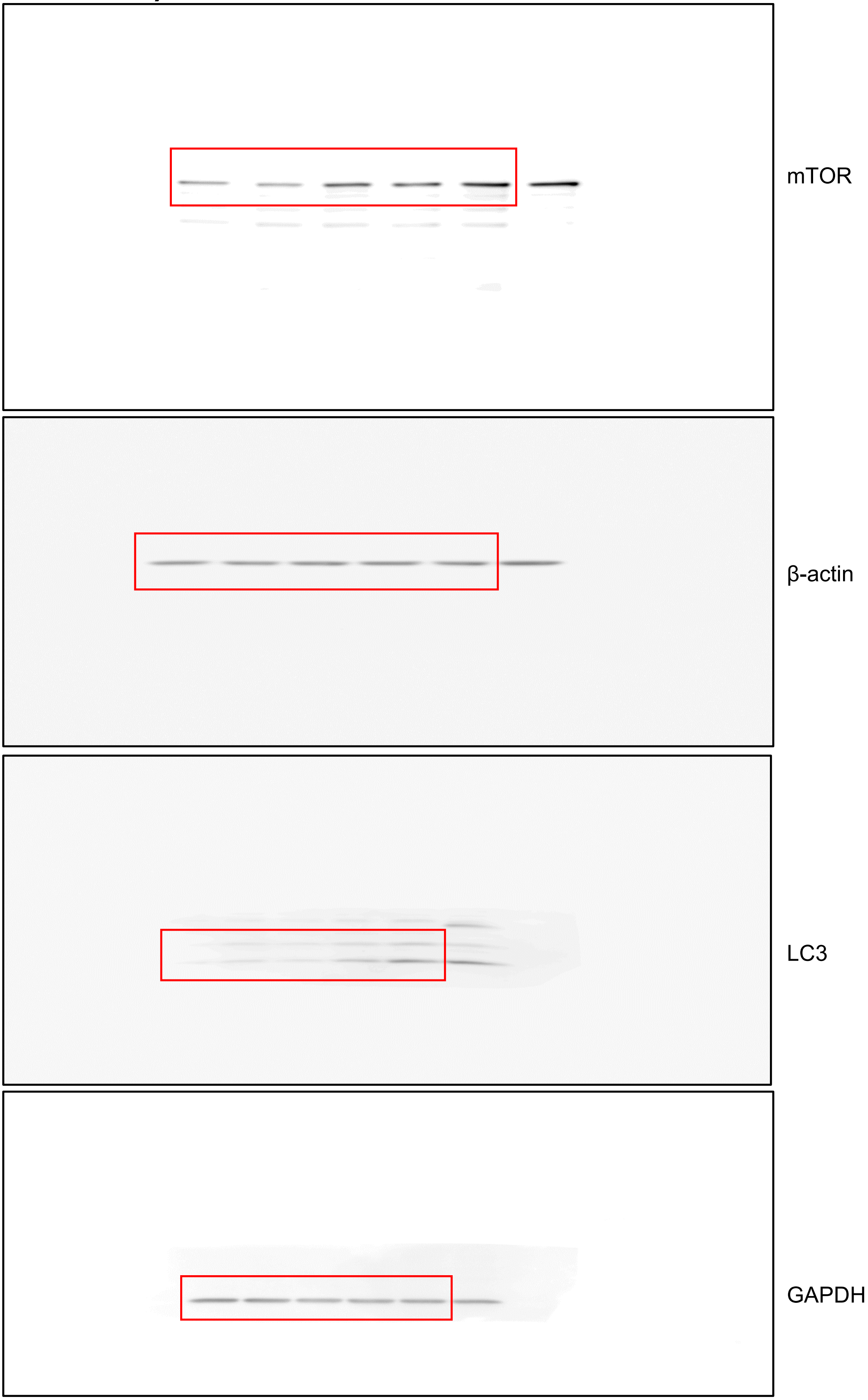

Fig. 3C repeated

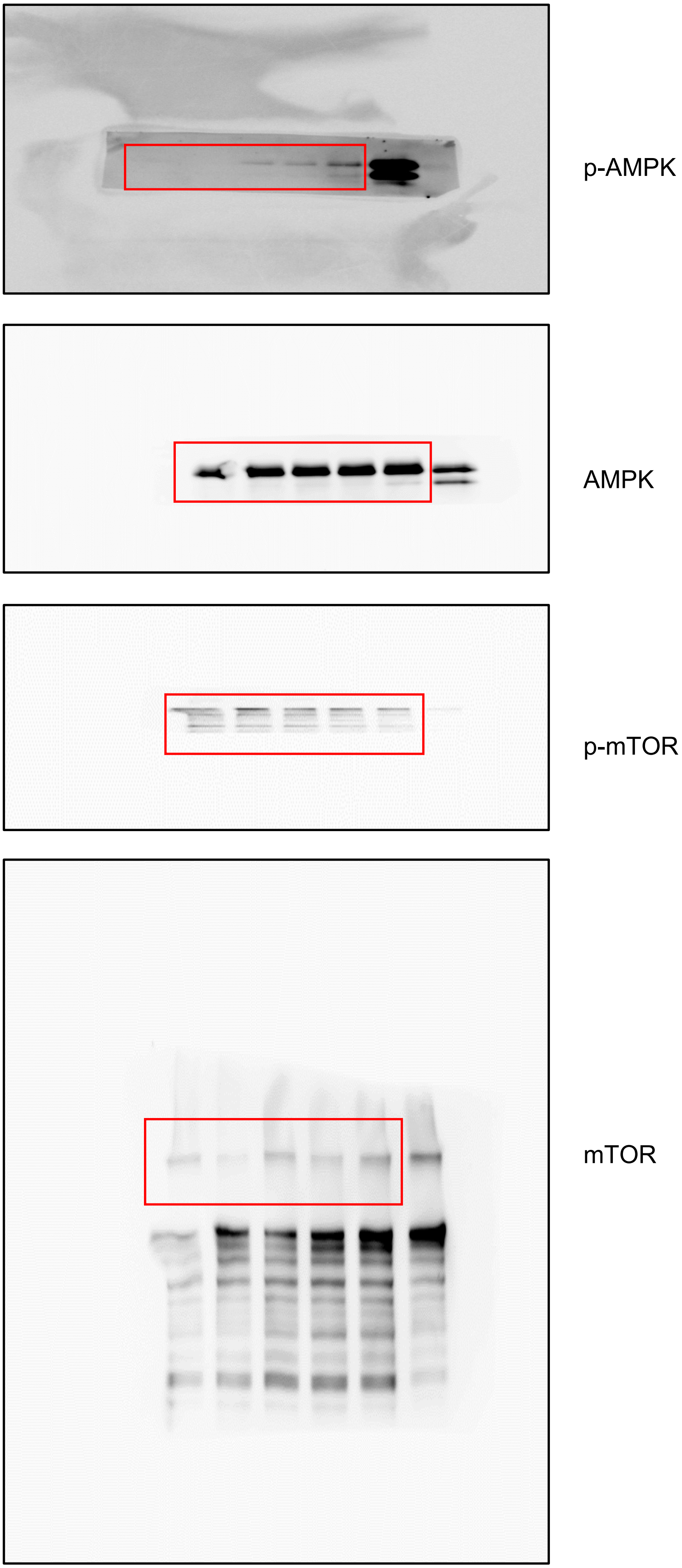

Fig. 3C repeated (continued)

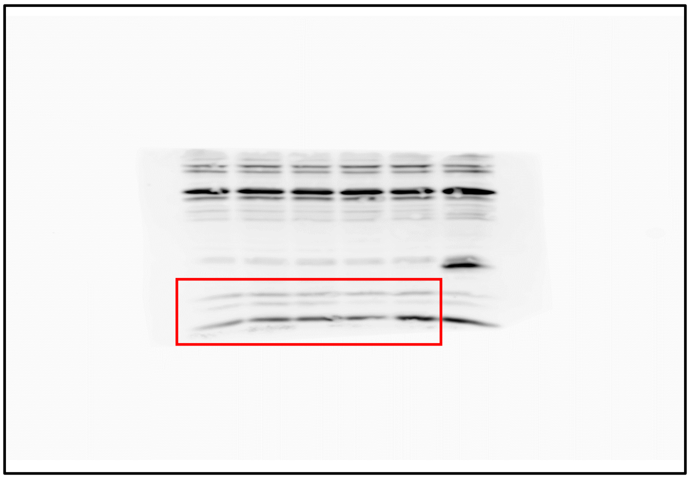

LC3

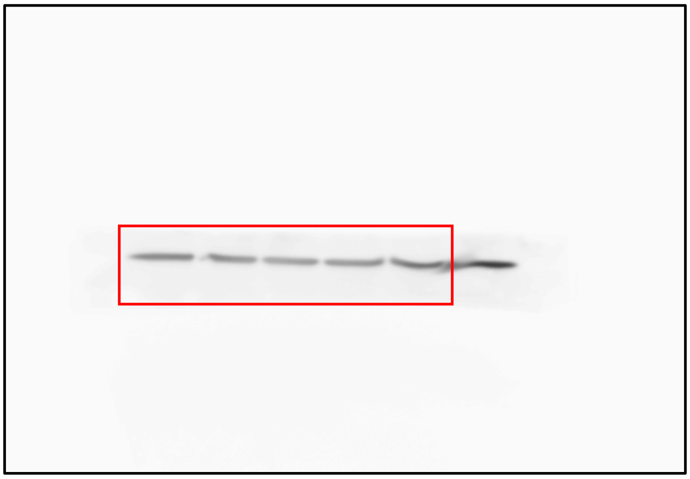

GAPDH

Fig. 4A

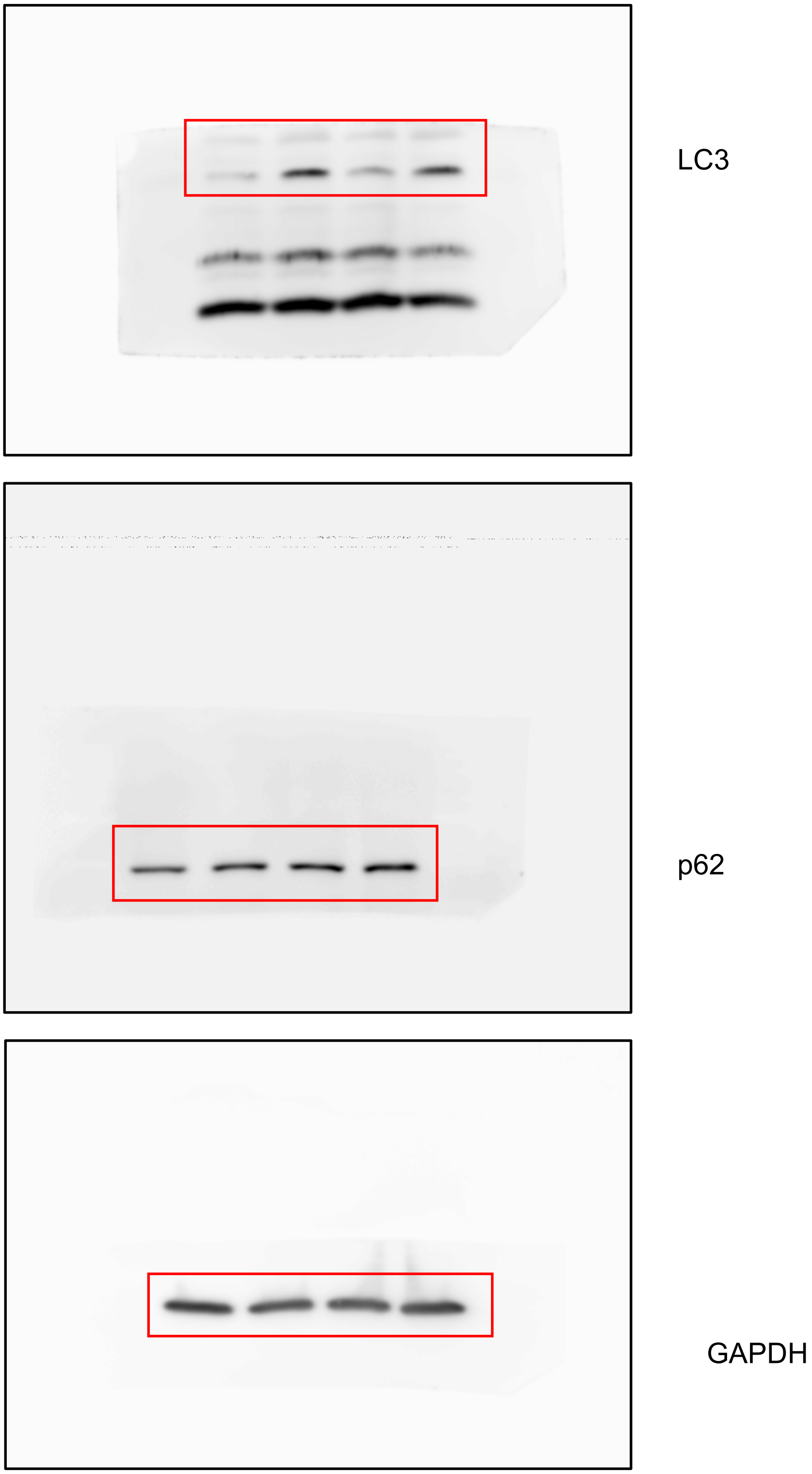

Fig.4B (continued)

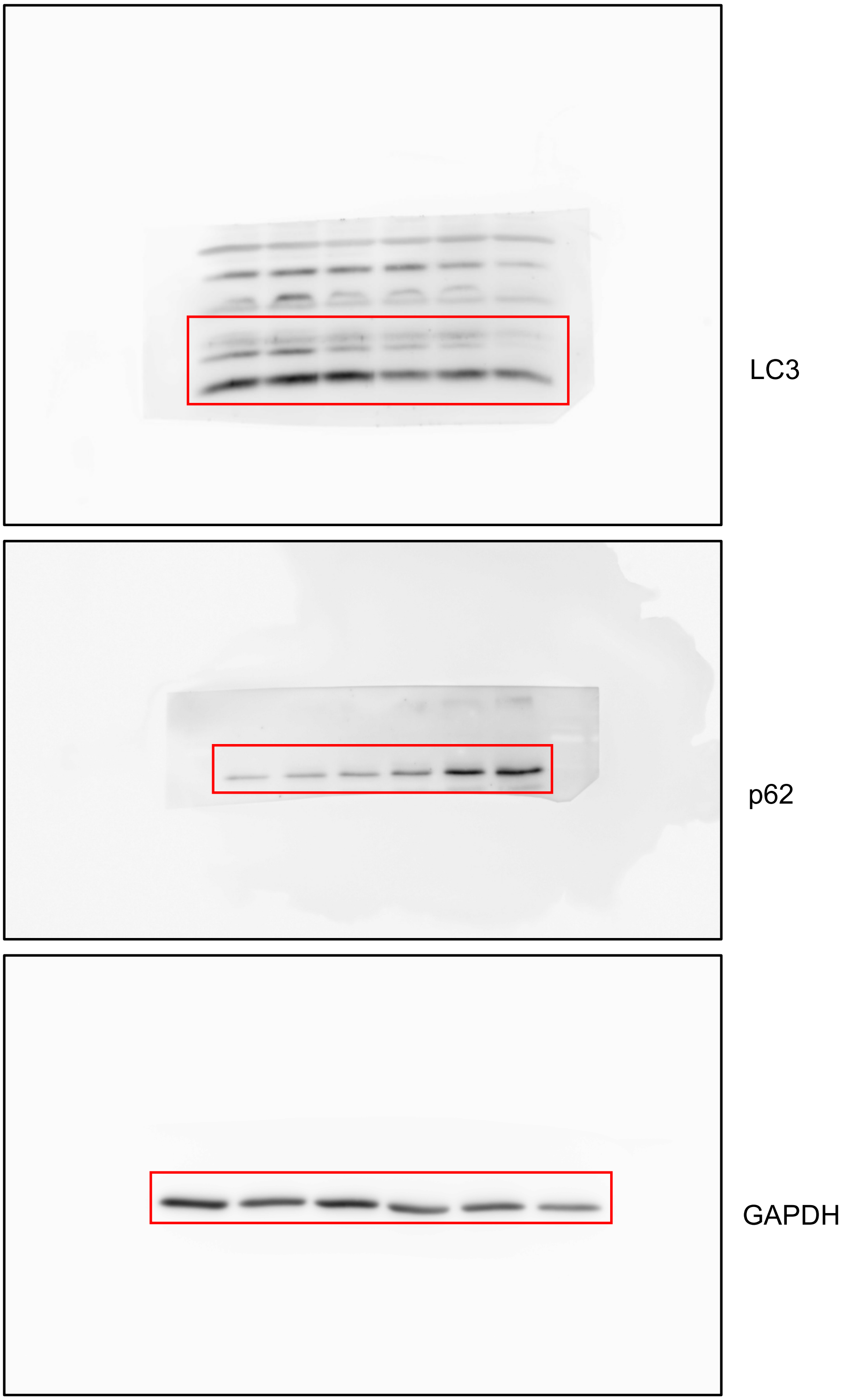

Fig. 4A repeated

Although the order of the samples differs from that in the manuscript, the samples were obtained under the same conditions.

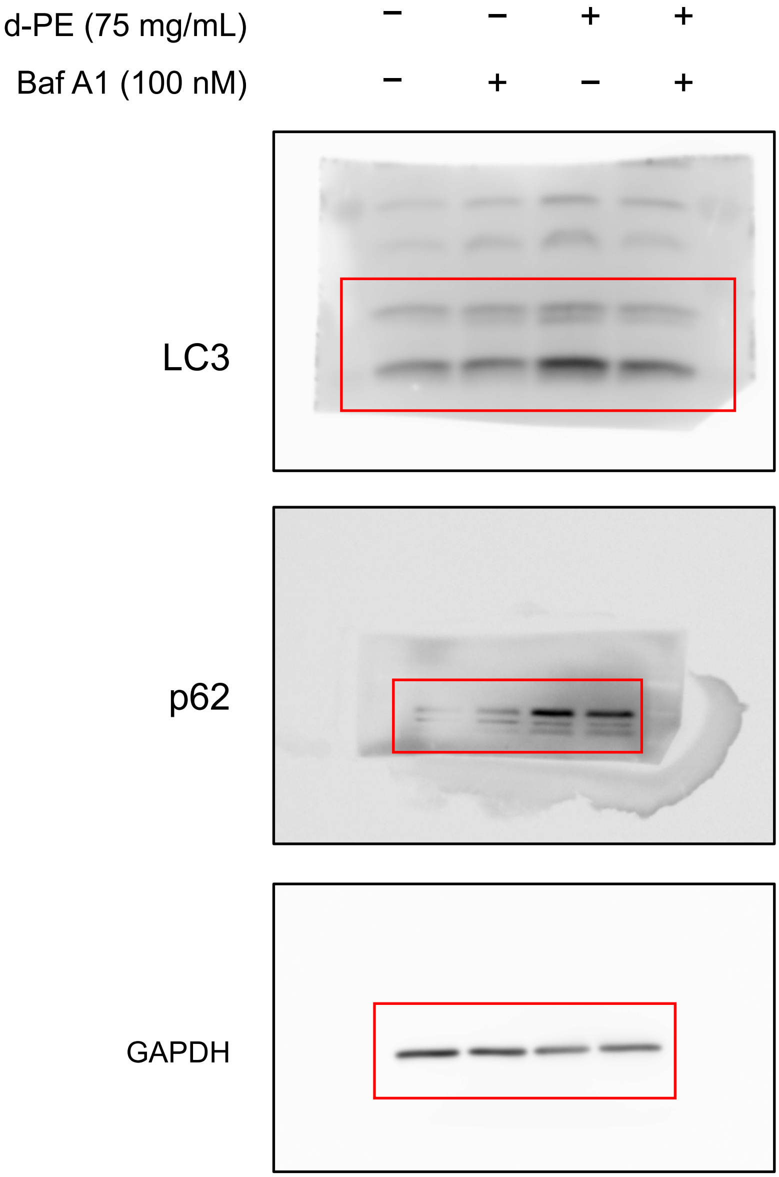

Fig. 4B repeated

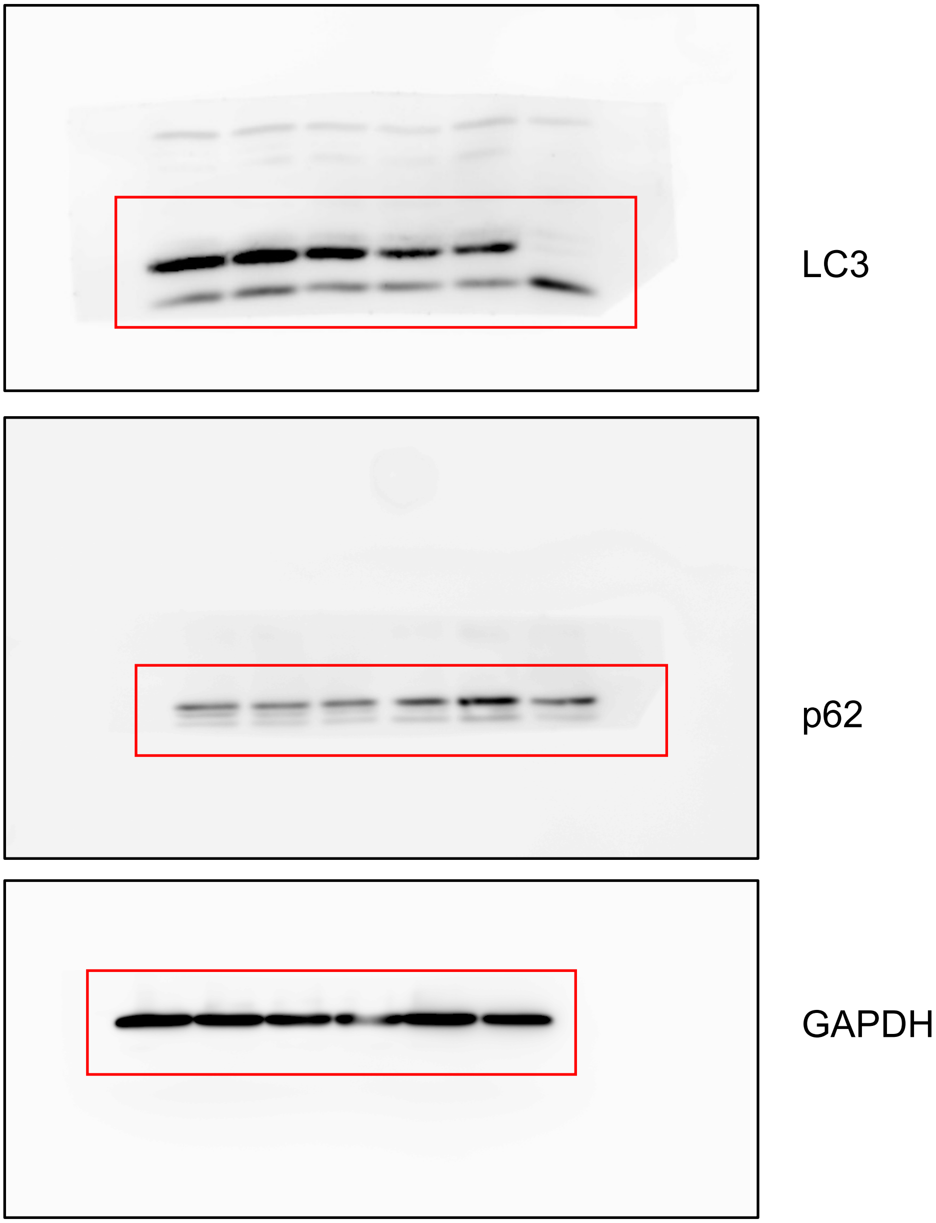

Supplement: Supplementary file 1 — Supplementary Information. [file 41598_2024_74800_MOESM1_ESM.pdf]
